# Snap-freezing in the Field: Effect of Sample Holding Time on Performance of Bactericidal Assays

**DOI:** 10.1093/icb/icac007

**Published:** 2022-03-16

**Authors:** Natalie M Claunch, Cynthia J Downs, Laura A Schoenle, Samantha J Oakey, Teresa Ely, Christina Romagosa, Christopher W Briggs

**Affiliations:** School of Natural Resources and Environment, University of Florida, Gainesville, FL 32601, USA; Department of Wildlife Ecology and Conservation, University of Florida, Gainesville, FL 32611, USA; Department of Environmental Biology, State University of New York College of Environmental Science and Forestry, Syracuse, NY 13210, USA; Office of Undergraduate Biology, Cornell University, Ithaca, NY 14850, USA; College of Veterinary Medicine, University of Georgia, Athens, GA 30602, USA; Golden Gate Raptor Observatory, Golden Gate National Parks Conservancy, Sausalito, CA 94965, USA; Department of Wildlife Ecology and Conservation, University of Florida, Gainesville, FL 32611, USA; Department of Biology, Colgate University, Hamilton, NY 13346, USA

## Abstract

Comparative analyses in biology rely on the quality of available data. Methodological differences among studies may introduce variation in results that obscure patterns. In the field of eco-immunology, functional immune assays such as antimicrobial capacity assays are widely used for among-species applications. Sample storage time and animal handling time can influence assay results in some species, but how sample holding time prior to freezing influences assay results is unknown. Sample holding time can vary widely in field studies on wild animals, prompting the need to understand the implications of such variation on assay results. We investigated the hypothesis that sample holding time prior to freezing influences assay results in six species (*Leiocephalus carinatus, Iguana iguana, Loxodonta africana, Ceratotherium simum, Columba livia*, and *Buteo swainsoni*) by comparing antibacterial capacity of serum with varying processing times prior to snap-freezing. Blood was collected once from each individual and aliquots were placed on ice and assigned different holding times (0, 30, 60, 180, and 240 min), after which each sample was centrifuged, then serum was separated and snap-frozen on dry ice and stored at −80ºC for 60 days prior to assaying. For each aliquot, we conducted antibacterial capacity assays with serial dilutions of serum inoculated with *E. coli* and extracted the dilution at 50% antibacterial capacity for analysis. We found a decrease in antibacterial capacity with increased holding time in one of the six species tested (*B. swainsoni*), driven in part by complete loss of antibacterial capacity in some individuals at the 240-min time point. While the majority of species’ antibacterial capacity were not affected, our results demonstrate the need to conduct pilot assays spanning the anticipated variation in sample holding times to develop appropriate field protocols.

## Introduction

Large-scale comparative analyses across species are critical to advancing the fields of organismal biology (Nakagawa and Santos 2012). Meta-analyses rely on the quality of available data; namely, it is critical that results from individual studies and laboratory procedures are reported and comparable across individual labs and species examined to draw conclusions from synthesizing these data in meta-analyses (Fanson et al. 2017). Eco-immunology requires comparative analyses to test hypotheses about the evolution of immune function (Brock et al. 2014; Schoenle et al. 2018). In the field of eco-immunology, researchers employ functional immune assays to assess immune investment in a variety of species and optimization of these assays for small amounts of blood increase the application in small species (Liebl and Martin 2009; French and Neuman Lee 2012; Downs and Stewart 2014; Jacobs and Fair 2016; Albert-Vega et al. 2018). As such, we are gaining baseline immune function information in many taxa (Matson et al. 2006; Millet et al. 2007), allowing the testing of trade-off hypotheses among immune function and species’ life history strategies and environments (e.g., Tieleman et al. 2005; Schneeberger et al. 2013; Heinrich et al. 2016; Refsnider et al. 2021). It is thus important to account for sources of variation in eco-immunological data to reduce statistical noise within studies to enable sound interpretation and use of functional immune data in future meta-analyses.

Variation in sample collection and treatment can influence the resulting interpretations of functional immunity. Differences in handling time of animals can induce physiological responses that influence assay results, such as decreases in antimicrobial capacity in birds with increased handling time (Matson et al. 2006; Becker et al. 2019). After collection, sample-handling can also influence functional immune assay results. Storage temperature influences activity of the complement pathway, an important component of antimicrobial immunity, in humans (O'Shaughnessy et al. 2012; Park et al. 2018). Repeated thawing and freezing of samples, which is often necessary for use of samples across multiple assays, can influence assay results as well (Liebl and Martin 2009; but see Hegemann et al. 2017). There is notable variation among taxa on the influence of sample treatment on assay results. No effect of time in the freezer was observed in several taxa, including some bats (Schneeberger et al. 2013; Becker et al. 2017), feliform carnivores (Heinrich et al. 2016; Flies et al. 2016), and common snapping turtles (*Chelydra serpentina*; Beck et al. 2017). Bird antimicrobial capacity appears to be sensitive to both animal handling and sample time in the freezer (Liebl and Martin 2009; Becker et al. 2019), but not all bird species lose antibacterial capacity with freezer time (Schneeberger et al. 2013; Jacobs and Fair 2016). Sample treatment prior to freezer storage may influence some of this observed variation.

Field collection of blood samples introduces an additional difficulty of standardizing sample processing and holding time that may not be as pervasive in captive animal studies, where processing equipment and storage facilities are often on-site. The reported holding time for blood samples prior to processing (centrifugation and freezing) spans from initiating assays immediately after collection; (e.g., no freezing; Matson et al. 2006; Liebl and Martin 2009), processing immediately after collection (e.g., Claunch et al. 2021), to processing 24 h after collection (e.g., Heinrich et al. 2016). Sometimes holding time of samples is unreported or vague (e.g., “on the same night,” “upon return to laboratory,” “within “x” hours”), implying variation in holding time between samples from different individuals. In field-based studies, the variation in blood sample holding time is influenced by several factors. First, the unpredictable nature of capturing animals may result in some samples with longer holding times simply because those animals were captured earlier. Second, the ability to process blood samples is often limited by the access to power to operate a centrifuge at remote field sites. Finally, even if centrifugation is possible, sample freezing is limited by the ability to obtain and transport adequate volumes of dry ice or liquid nitrogen to maintain frozen samples prior to returning to the lab for final sample storage. Often, whole blood samples are stored on ice until returning for processing to the laboratory (e.g., Jacobs and Fair 2016; Titon et al. 2018). Some components of blood serum involved in bacterial killing, such as proteins (e.g., complement and other antimicrobial peptides) and lectins, may deteriorate over time and during storage above certain temperatures (Hatten et al. 1973; Petrakis 1985; Laursen and Nielson 2000; Matson et al. 2006; O'Shaugnessy et al. 2012). Thus, it is possible that variation in sample holding time prior to freezing may influence the performance of serum in antibacterial assays.

Variation in sample holding time may introduce noise to immune function data that could mask or prevent interpretation of the central hypotheses within a study. This issue could compound to affect comparisons of data across studies and interpretation of meta-analyses. To understand how field-relevant variation in blood sample processing time influences interpretation of assays, we assessed the effects of sample holding time prior to freezing on the antibacterial capacity of serum using aliquots of whole blood from individuals of six species varying in size, life history, and taxonomic class.

## Methods

### Blood sampling

Wild reptiles were captured by pole and lasso. Adult green iguana (*Iguana iguana*) were captured in Key Largo, Florida, USA and bled from the caudal vessels with a needle rinsed with sodium citrate; anesthesia was not used. Adult Northern curly-tailed lizards (*Leiocephalus carinatus*) were captured in Indian Rocks Beach, Florida, USA, and following anesthesia with Isoflurane, were bled from cardiac puncture using a needle rinsed with sodium heparin before euthanasia. Captive adult African elephants and white rhinoceros (*Loxodonta africana* and *Ceratotherium simum*) were sampled at ZooTampa in Tampa, Florida, USA by trained veterinary staff using a heparin coated needle. Samples were taken from an ear vein in each species, and no animals were under sedation at time of sampling. We sampled captive rock pigeons (*Columba livia*) at Golden Gate Raptor Observatory in Sausalito, California, USA. Up to 0.3 cc of blood was drawn from the femoral vein of each individual using a syringe. Blood was pooled across three individuals in lithium heparinized tubes to obtain sufficient sample to aliquot it. Swainson's hawk (*Buteo swainsoni*) nestlings that were at least 21-days old were sampled in Butte Valley, California, USA. We extracted up to 0.75 mL of blood from the brachial vein in a lithium heparinized tube.

### Blood sample treatment protocol

Initially we collected a single 0.5–2 mL sample of blood from each animal using syringes rinsed with anticoagulant or into tubes coated with anticoagulant (Table 1). We then immediately separated this whole blood into 5 aliquots—these tubes did not contain anticoagulant. Four of the whole blood aliquots were placed into a cooler on ice, and we immediately centrifuged (rpm) the one remaining whole blood aliquot to separate serum from packed cells. After pipetting serum into new tubes, the serum samples were snap-frozen on dry ice. The four whole blood aliquots were removed from the cooler for centrifugation and serum separation at 30, 60,180, and 240 min after the first sample was processed prior to snap freezing on dry ice as above (time-to-freeze). Samples were transported to a −80°C freezer for storage. Because overall antimicrobial activity may decrease with storage time in the freezer (e.g., Liebl and Martin 2009), we standardized freezer storage time for 62 days (+/- 3) before running antibacterial activity assays; shorter storage time was not possible due to scheduling constraints. We followed this protocol for all six species (Table 1).

**Table 1:** Details on numbers of each species sampled for blood and dilution of serum and plasma used in antibacterial capacity assays

Species	Number of Individuals	Anticoagulant	Ultracold time prior to assaying	Number of dilutions	Dilution range
Green iguana	7	needle rinsed with sodium citrate	68 days	5	0.09375–0.005859
Curly-tailed lizard	8	needle rinsed with sodium heparin	61 days	5	0.0625–0.003906
Swainson's hawk	10	tube coated with lithium heparin	60–65 days	5	0.1875–0.03125
Rock pigeon	18 (6 pools of 3)	tube coated with lithium heparin	59 days	5	Raw–0.0625
African elephant	5	needle rinsed with sodium heparin	60–62 days	6	0.375–0.005859
Southern white rhinoceros	5	needle rinsed with sodium heparin	60–61 days	6	0.375–0.005859

### Antibacterial capacity assay

To assess antibacterial capacity, we performed a functional assay that measures the growth of *Escherichia coli* in the presence of blood serum using a procedure adapted from Schoenle et al. (2020, Downs et al. 2021). We chose to use *E. coli* as our microbe because it is commonly used and pervasive in eco-immunology studies across taxa (e.g., Becker et al. 2019). Each time-to-freeze aliquot from each individual was thawed on ice, vortexed, then plated in triplicate in a sterile 96-well plate. We randomly assigned each aliquot's position on the plate such that the position of processing times varied across plates. We diluted samples with 1M sterile Phosphate Buffered Saline (PBS; Lonza 12001–678; see Table 1 for dilution information). The volume of each diluted sample equaled 18 μL, regardless of dilution. Dilutions differed among species to ensure we captured the full range of antibacterial capacity (0–100%) within a species’ dilution series (Table 1); we determined these values from pilot data generated from serial dilutions of pooled samples from each species. Across all assays, a serial dilution of cow serum in PBS was included as a standard (4 dilutions from 0.03125 to 0.003906) plated in triplicate. Additionally, three wells of negative controls (absence of serum and bacteria to monitor potential contamination of reagents) and six wells of positive controls (absence of serum to quantify maximum growth in broth) were assigned to each plate.

We added 2 μL of a solution of 10^4^ colony-forming units of *E. coli* (ATCC 8739) diluted in PBS to all wells except negative controls, such that the volume of each well equaled 20 μL. Plates are vortexed for 1 min at 700 rpm, incubated at 37°C for 30 min, and vortexed again. Then, 125 μL of sterile tryptic soy broth (TSB, Sigma-Aldrich T8907) was added to all wells and vortexed at 300 rpm for 1 min. The plate was read at 300 nm in a microspectrophotometer (0 h) and incubated at 37°C for 12 h, after which it was vortexed at 300 rpm for 1 min and read again at 300 nm to quantify bacterial growth.

Antibacterial capacity was calculated similar to Claunch et al. (2021). First, the 0 h optical density was subtracted from the 12 h optical density for all wells. Sample replicates with greater than 10% variation in 12 h optical density were removed from calculations, the remaining replicates are averaged for further calculation. The resulting 12 h difference from each sample aliquot's dilution was then subtracted from the average 12 h difference in optical density of positive control wells, and finally divided by the average 12 h difference in optical density of positive control wells to calculate % bacterial growth inhibited (antibacterial activity). We created a curve from the antibacterial activity values from each sample dilution series.

### Statistics

**Fig. 1:**
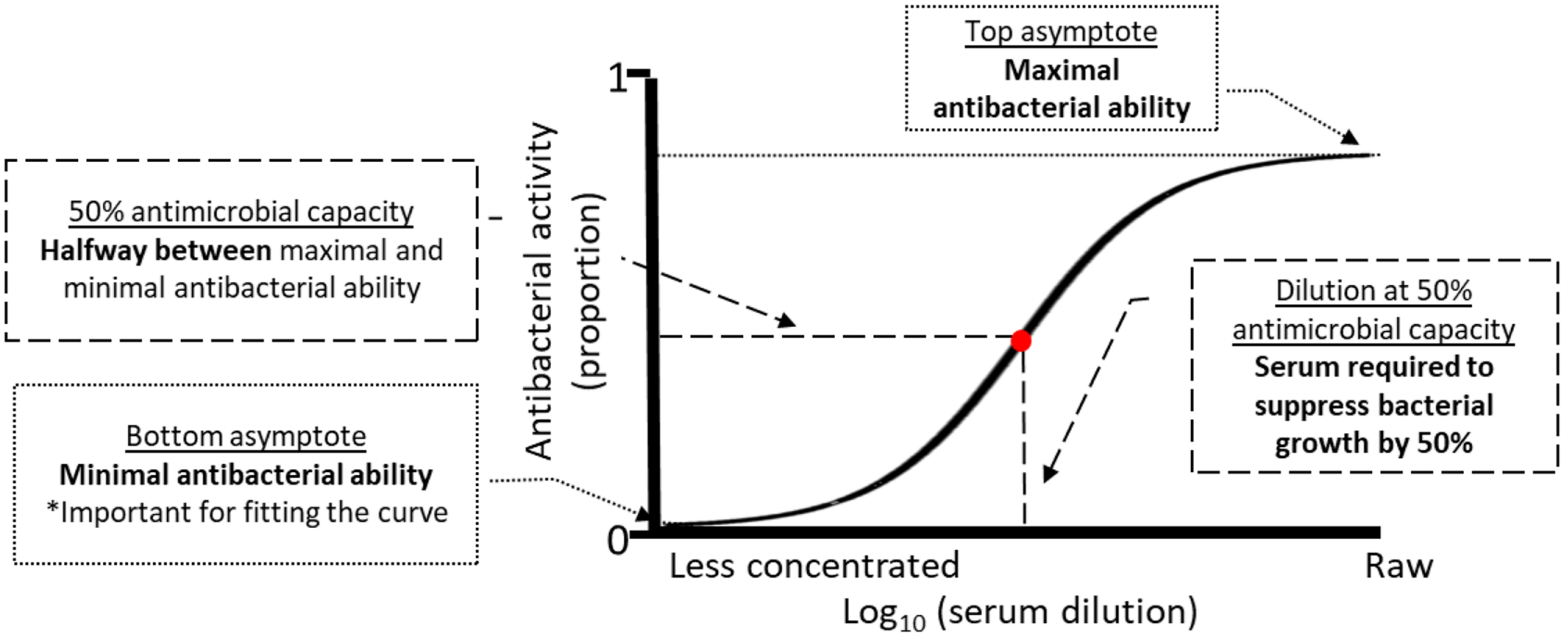
Hypothetical non-linear curve for antimicrobial activity of serum for a single individual. The dilution at 50% antimicrobial capacity is extracted from each curve to enable comparisons of sample treatment within each individual (adapted from Downs et al. 2021).

Following Downs et al. (2021), we fit 5-parameter logistic regression growth curves to the dilution curves for each sample using package nplr (Commo and Bot 2016) to determine antibacterial capacity for each sample (Fig. 1). To aid in curve-fitting, we log10-transformed serum concentrations (i.e., the dilutions) and convert the antibacterial ability from a % to a proportion. Curves could only be fit to values between 0 and 1. Thus, antibacterial capacity values > 100% were forced to a random value between 99 and 100 (8.3% of sample dilutions) and antibacterial capacity values < 0% (14.9% of sample dilutions) were forced to a random value between 0 and 1 before conversion to proportions. We extracted the log-transformed 50% antibacterial capacity (i.e., the value halfway between top and bottom asymptotes) to use as our response variable (Fig. 1).

To evaluate the effects of time-to-freeze on antibacterial capacity of serum among species, we ran a linear mixed effects model in R (R Core Team, 2019) using the lme4 package (Bates et al. 2015). The response variable was the log-transformed 50% antibacterial capacity, and the model included fixed effects of time-to-freeze as a continuous variable, species as a factor, and an interaction between the two. To account for repeated sampling of individuals we included animal ID as a random effect. Where the interaction was significant, individual linear mixed effects models were run for each species to elucidate within-species effects. Type II Wald Chi-Square tests were used to assess significance of each fixed effect of interaction. We set α = 0.05 for all analyses. Data are available at https://github.com/nmclaunch/snap_freezing_field.

### Permits

Sampling was conducted in accordance with the following protocols and permits: Reptiles- UF IACUC 201,709,774, EVER 2018-SCI-0036; Mammals- USF IACUC T IS00004920; Rock pigeons- CA_GOGA_Ely_Pigeons_2021.A3; Swainson's hawks-Hamilton College IACUC 19-R-5, California Scientific Collecting permit 007333 USGS banding permit 24019.

## Results

**Fig. 2:**
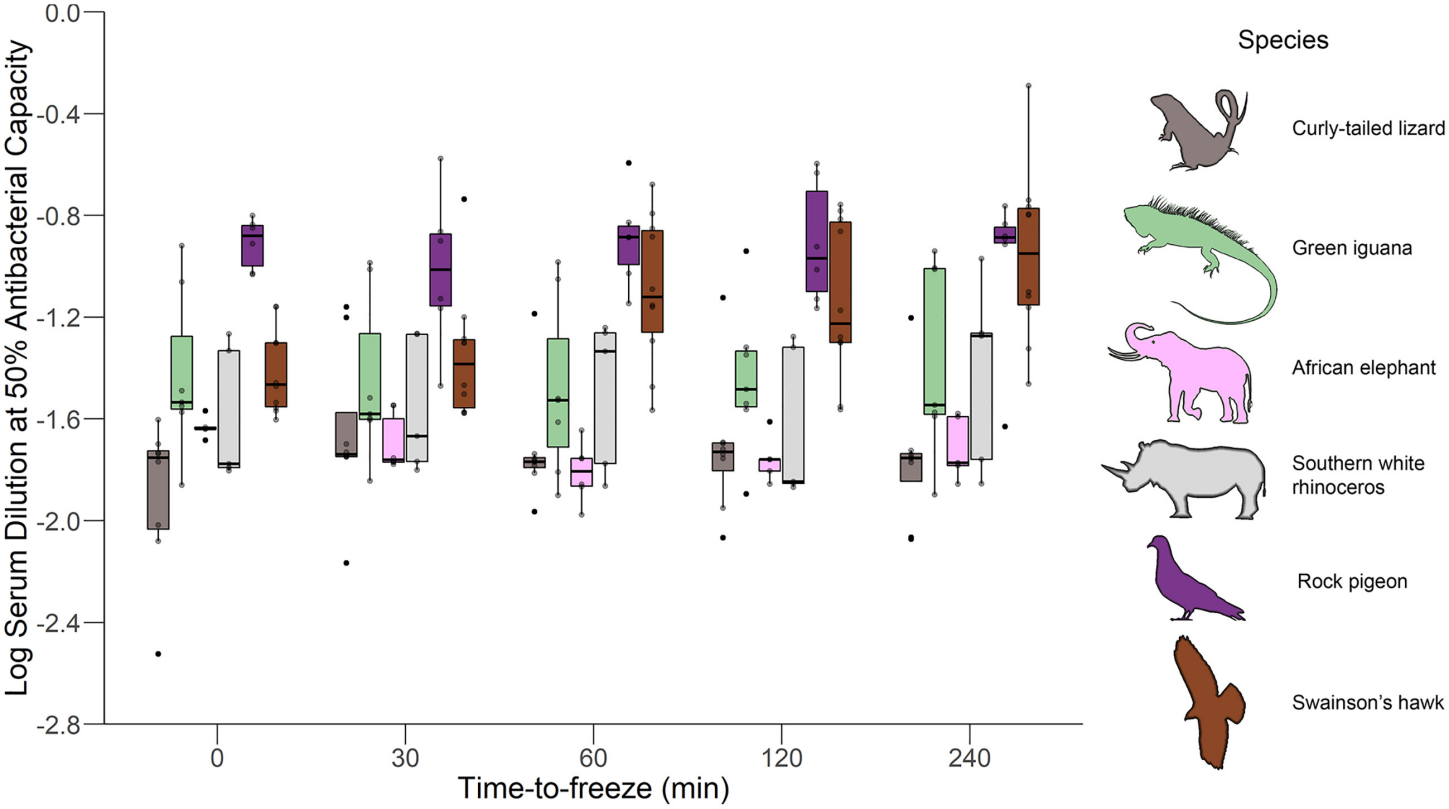
Boxplots showing log-transformed dilution of serum corresponding to 50% antibacterial capacity for each species at each aliquot's time-to-freeze (time on ice before centrifugation, serum separation, and snap-freezing). A 0 value for dilution corresponds to undiluted or raw serum. Time was a continuous variable in analyses. Swainson's hawks exhibited increases in the amount of serum required to maintain 50% antibacterial capacity with increases in sample holding time, resulting in decreased antibacterial capacity with time before snap-freezing of serum. Holding time before processing and freezing did not influence antibacterial capacity in the other species.

Time-to-freeze interacted with species to influence antibacterial capacity (χ^2^_1,3_ = 20.909, *P* < 0.001). The main effect of species was significant (χ^2^_1,3_ = 31.232, *P* < 0.001), as was the main effect of time-to-freeze (χ^2^_1,3_ = 19.310, *P* < 0.001), where slightly more serum was required to achieve 50% killing at longer time-to-freeze (0.0001968 +/− 0.00039 Std Error). Individual species analyses revealed that these main effects were primarily driven by variation in Swainson's hawk samples, which demonstrated decreased antibacterial capacity with time-to-freeze (i.e., more serum required at longer freeze times; 0.002 +/− 0.0005; χ^2^ = 20.582, *P* < 0.001), with four of the ten individuals showing complete loss of antibacterial capacity at the longest time-to-freeze (240 min). The other species’ antibacterial capacities were not significantly influenced by time-to-freeze (rock pigeon: χ^2^ = 0.024, *P* = 0.878; elephant: χ^2^ = 0.0051, *P* = 0.943; rhinoceros: χ^2^ = 0.412, *P* = 0.521; iguana: χ^2^ = 1.024, *P* = 0.312; curly tailed lizard: χ^2^ = 0.308, *P* = 0.579; Fig. 2).

## Discussion

We found that the amount of holding time before the centrifugation and snap freezing of blood samples (time-to-freeze) influenced antibacterial capacity in samples from Swainson's hawks, but not from the other species tested. Our results add to those of other studies which found sensitivity of antibacterial capacity to sample handling may be species-specific. For example, in one passerine bird (house sparrow, *Passer domesticus*), antibacterial capacity decreased with freezer storage time (Liebl and Martin 2009); whereas antibacterial capacity was not influenced by time in freezer or time before processing in a galliform (*Gallus gallus*) and two other passerines (*Myiarchus cinerascens* and *Sialia mexicana*; Jacobs and Fair 2016). Other non-avian species did not lose antibacterial capacity with sample storage time (mammals: Schneeberger et al. 2013; Heinrich et al. 2016; Flies et al. 2016; Becker et al. 2017; Becker et al. 2019; reptile: Beck et al. 2017). Combined with our observation that holding time influenced antibacterial capacity in Swainson's hawks, but not pigeons, these results suggest that antibacterial components in certain bird serum samples may be especially sensitive to handling and storage conditions.

The fact we observed time-to-freeze effects in Swainson's hawks, but did not observe similar decreases in rock pigeon antibacterial capacity is not entirely surprising, given that different bird species demonstrate inherently different antibacterial capacity (Matson et al. 2006; Millet et al. 2007). The primary components associated with bacterial killing in animal serum include complement (Merle et al. 2015), antibodies (Matson et al. 2006), lectins (Laursen and Nielsen 2000), and may also include other antimicrobial peptides and proteins (Zimmerman et al. 2010). Differences in the presence of these components and their associated sensitivities to handling among species may influence effects of hold-time on antibacterial capacity among species. For example, complement proteins and activation pathways can vary by species (Koppenheffer and Russell 1986) and are thermally sensitive; this thermal-sensitivity appears to vary among species (Hatten et al. 1973). Compared to mammals, birds and reptiles have less complex complement systems (Nakao and Somamoto 2016), which may render them more sensitive to loss of functionality (reviewed in Becker et al. 2019). However, there are still major gaps in understanding of bird and reptile complement components and systems which preclude in-depth discussion and comparison among species (Dodds and Matsushita 2007; Zimmerman et al. 2010). Apart from complement, some species exhibit potent antimicrobial peptides (e.g., crocodiles; Preecharram et al. 2008; Bishop et al. 2015). If certain antimicrobial components can maintain potent antimicrobial capacity while the capacity of others are reduced by cool handling and storage, some taxa may retain similar functional antibacterial capacity of serum across a range of sample treatments.

As Swainson's hawks were sampled as nestlings, it is also possible that components of nestling immunity are more sensitive to sample handling. Nestlings may have different immune capacity than adults (e.g., lack of antibodies from prior pathogen exposure), and may rely instead on immune components that are more sensitive to hold time effects, though this remains to be tested. Age-related immunocompetence of microbiocidal ability has been documented in zebra finches (*Taeniopygia guttata*), where increased microbiocidal ability was observed in some juvenile animals (Noreen et al. 2011), but sensitivity of this ability to sample handling was not assessed. Comparisons of nestlings through adults will be necessary to resolve whether reduced microbiocidal capacity at longer time-to-freeze is age or species specific within Swainson's hawks.

Though our sampling was limited to six species, and our data do not allow us to draw conclusions on phylogenetic patterns, our results reiterate that protocols should be optimized and verified before applying to new taxa. This optimization process should thoroughly investigate potential sources of variation such as sample holding time. Limitations on assay interpretation may be present in some groups, in our case Swainson's hawk nestlings, that warrant standardization of sample holding time that may not be necessary with others. It is important to note that we only evaluated sample holding time effects on one microbe, *E. coli*, and our results may not reflect the effect of sample holding time on other microbes, as different components of serum are employed in antimicrobial activity against various microbes (e.g., Pulendran et al. 2001) resulting in differences in antimicrobial capacity (French and Neuman-Lee, 2012). In any case, the best practice may be to record holding and processing times for each sample and include these values as a covariate in analyses to control for its influence on assay variability. Finally, reporting sample holding times with more precise values (e.g., including standard deviation), will allow better interpretation of antibacterial capacity data in future meta-analyses.

## Notes

From the symposium “Ecoimmunology: what unconventional organisms tell us after two decades” presented at the annual meeting of the Society for Integrative and Comparative Biology virtual annual meeting, January 3–February 28, 2022.
